# Supplementary material for: The craniomandibular anatomy of the early archosauriform Euparkeria capensis and the dawn of the archosaur skull
Source: R Soc Open Sci. 2020 Jul 29;7(7):200116. doi: 10.1098/rsos.200116 (PMC7428278; doi:10.1098/rsos.200116)
Supplement: Figure S1 - AMNH 19351.pdf [file rsos200116supp1.pdf]

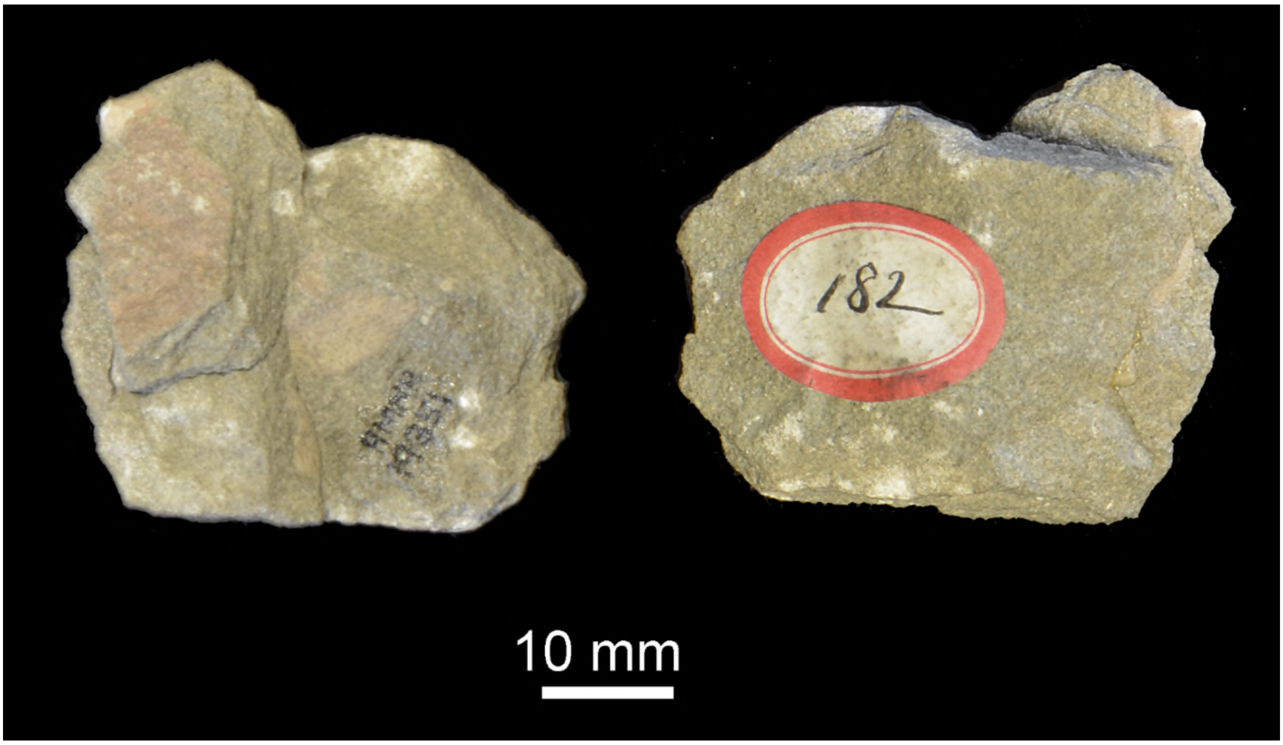

Supplementary Figure 1. Specimen AMNH 19351, attributed to *Euparkeria capensis*, showing opposite sides of block left and right.
