# Supplementary material for: The craniomandibular anatomy of the early archosauriform Euparkeria capensis and the dawn of the archosaur skull
Source: R Soc Open Sci. 2020 Jul 29;7(7):200116. doi: 10.1098/rsos.200116 (PMC7428278; doi:10.1098/rsos.200116)
Supplement: Table S1 - Measurements.pdf [file rsos200116supp2.pdf]

Table S1. Key cranial measurements for *Euparkeria capensis*.

**Dimension**	**Specimen**	**Measurement (mm)**
Maximum length of skull from posterior of occipital condyle to tip of rostrum (=occipitorostral length)	SAM-PK-5867	61.5
Estimated occipitorostral length	SAM-PK-6047A	73
Maximum dorsoventral height of skull	SAM-PK-5867	37 (to bottom of mandible), 30 (to bottom of jugal)
Maximum mediolateral width of skull	SAM-PK-5867	33 (across squamosals)
Width of skull level with anteroposterior midpoint of external nares	SAM-PK-5867	10
Maximum dorsoventral height of naris	SAM-PK-6047A (left)	10
Maximum anteroposterior length of naris	SAM-PK-6047A (left)	11
Minimum anteroposterior length of bar separating external naris and antorbital fenestra (=minimum “width” of bar in lateral view)	SAM-PK-5867 (right)	9
Maximum anteroposterior length of external antorbital fenestra	SAM-PK-5867	22
Maximum dorsoventral height of external antorbital fenestra	SAM-PK-5867 (right)	14
Maximum anteroposterior length of internal antorbital fenestra	SAM-PK-5867 (right)	20
Maximum dorsoventral height of internal antorbital fenestra	SAM-PK-5867 (right)	11
Minimum anteroposterior length of antorbital bar (=minimum “width” of bar)	SAM-PK-5867 (right)	4
Maximum dorsoventral height of orbit	SAM-PK-5867 (right)	21
Maximum anteroposterior length of orbit	SAM-PK-5867 (right)	22
Length of skull anterior to orbit (anterior of orbital margin to tip of rostrum)	SAM-PK-5867 (right)	34 (as preserved)
Length of skull posterior to orbit (posterior of orbital margin to posterior of	SAM-PK-5867 (right)	19

postoccipital)		
Minimum anteroposterior length of postorbital bar (=minimum “width” of bar)	SAM-PK-5867 (right)	3
Anteroposterior length of lateral temporal fenestra at ventral margin	SAM-PK-5867 (right)	18
Anteroposterior length of lateral temporal fenestra at dorsal margin	SAM-PK-5867 (right)	6
Minimum anteroposterior length of lateral temporal fenestra	SAM-PK-5867 (right)	6
Maximum dorsoventral height of lateral temporal fenestra	SAM-PK-5867 (right)	20
Maximum mediolateral width of supratemporal fenestra	SAM-PK-5867 (right)	10
Maximum anteroposterior length of supratemporal fenestra	SAM-PK-5867 (right)	13
Minimum dorsoventral height of postorbital-squamosal bar (=minimum “width” of bar)	SAM-PK-5867 (right)	2
Minimum anteroposterior length of choana	SAM-PK-5867 (right)	14
Minimum mediolateral width of choana	SAM-PK-5867 (right)	4

Table S2. Key mandibular measurements for *Euparkeria capensis*.

**Dimension**	**Specimen**	**Measurement (mm)**

Mandibular length	SAM-PK-5867 (rhs)	86
Dorsoventral height of mandibular ramus at deepest point	SAM-PK-5867 (rhs)	14
Dorsoventral height of mandibular fenestra at deepest point	SAM-PK-5867 (rhs)	9
Anteroposterior length of mandibular fenestra at longest point	SAM-PK-5867 (rhs)	14
Length of mandible anterior to anteriormost point of mandibular fenestra	SAM-PK-5867 (rhs)	41
Length of mandible posterior to posteriormost point of mandibular fenestra	SAM-PK-5867 (rhs)	30
Height of mandible dorsal to dorsalmost point of mandibular fenestra	SAM-PK-5867 (rhs)	3
Height of mandible ventral to ventralmost point of mandibular fenestra	SAM-PK-5867 (rhs)	2
Dorsoventral height of Meckelian foramen at deepest point	SAM-PK-5867 (lhs)	7
Anteroposterior length of Meckelian foramen at longest point	SAM-PK-5867 (lhs)	19
Length of mandible anterior to anteriormost point of Meckelian foramen	SAM-PK-5867 (lhs)	43
Length of mandible posterior to posteriormost point of Meckelian foramen	SAM-PK-5867 (lhs)	17
Height of mandible dorsal to dorsalmost point of Meckelian foramen	SAM-PK-5867 (lhs)	4
Height of mandible ventral to ventralmost point of Meckelian foramen	SAM-PK-5867 (lhs)	2
