# Supplementary material for: The craniomandibular anatomy of the early archosauriform Euparkeria capensis and the dawn of the archosaur skull
Source: R Soc Open Sci. 2020 Jul 29;7(7):200116. doi: 10.1098/rsos.200116 (PMC7428278; doi:10.1098/rsos.200116)
Supplement: Phylogenetic anaysis and character mapping.pdf [file rsos200116supp3.pdf]

Phylogenetic analysis and character mapping

TNT code, trees resulting from phylogenetic analyses with support values, and mapping of palatal dention characters.

For further details of phylogenetic analysis, see main text.

TNT code used for phylogenetic analysis

##Main analysis

```
mxram5000;proc
sookias_2016.tnt;ccode+7+29+57+117+129+131+159+223+245+258+266+267+269+289+293+320+346+388;
Outgroup Youngina_capensis;hold10000;mu1000;bb;
```

#Saving strict consensus tree

```
taxname=;ne*;tsave* strict.tre;save248;tsave/;
```

#Character mapping tree

```
ttags-;ttags=;naked-;taxname=;apo[;tsave*strict_synapomorphies.tre;save*;tsave/;
```

##Bootstrap analysis

```
mxram5000;proc
sookias_2016.tnt;ccode+7+29+57+117+129+131+159+223+245+258+266+267+269+289+293+320+346+388;
Outgroup Youngina_capensis;hold10000;mu1000;bb;
```

#Saving bootstraps

```
ne*;ttags=;resample boot replications 1000 frequency GC from 248;taxname=;tsave*
strictbs.tre;save*;tsave/;
```

##Bremer support analysis

```
mxram5000;proc
sookias_2016.tnt;ccode+7+29+57+117+129+131+159+223+245+258+266+267+269+289+293+320+346+388;
Outgroup Youngina_capensis;hold10000;mu1000;bb;
```

#Saving Bremer support

sub20;bb;ne*;ttags=;bsupport;taxname=;tsave* strictbremer.tre;save*;tsave/;

####Conducting analyses without 'Turfanosuchus shageduensis'

mxram5000;proc
sookias_2016.tnt;ccode+7+29+57+117+129+131+159+223+245+258+266+267+269+289+293+320+346+388;
taxcode-59;Outgroup Youngina_capensis;hold10000;mu1000;bb;

#Saving strict consensus tree

taxname=;ne*;tsave* strictnoTS.tre;save248;tsave/;

##Bootstrap analysis

mxram5000;proc
sookias_2016.tnt;ccode+7+29+57+117+129+131+159+223+245+258+266+267+269+289+293+320+346+388;
taxcode-59;Outgroup Youngina_capensis;hold10000;mu1000;bb;

#Saving bootstraps

ne*;ttags=;resample boot replications 1000 frequency GC from 248;taxname=;tsave*
strictbsnoTS.tre;save*;tsave/;

##Bremer support analysis

mxram5000;proc
sookias_2016.tnt;ccode+7+29+57+117+129+131+159+223+245+258+266+267+269+289+293+320+346+388;
taxcode-59;Outgroup Youngina_capensis;hold10000;mu1000;bb;

#Saving Bremer support

sub20;bb;ne*;ttags=;bsupport;taxname=;tsave* strictbremernoTS.tre;save*;tsave/;

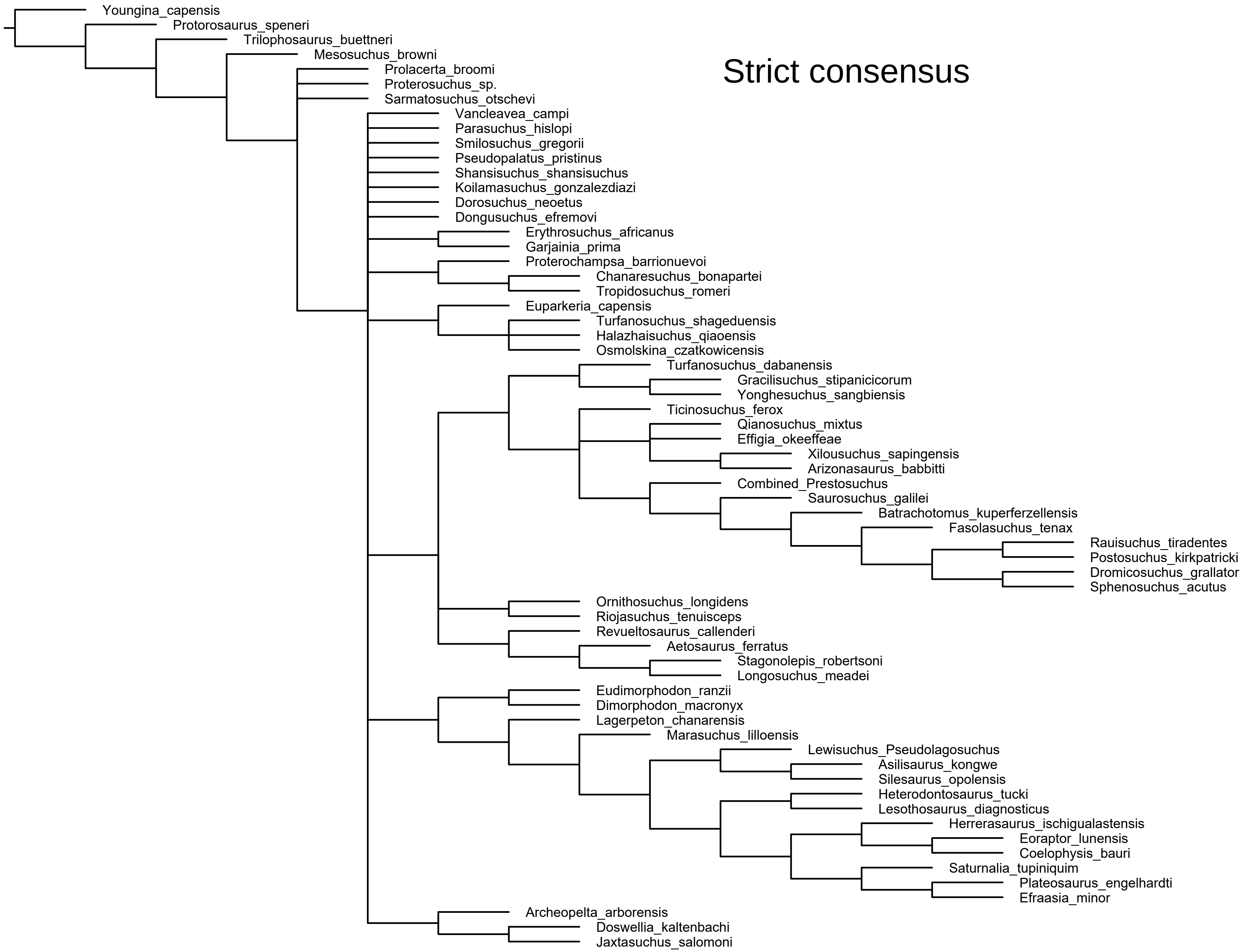
Strict consensus
Youngina_capensis
Protorosaurus_speneri
Trilophosaurus_buettneri
Mesosuchus_browni
Prolacerta_broomi
Proterosuchus_sp.
Sarmatosuchus_otschevi
Vancleavea_campi
Parasuchus_hislopi
Smilosuchus_gregorii
Pseudopalatus_pristinus
Shansisuchus_shansisuchus
Koilamasuchus_gonzalezdiazi
Dorosuchus_neoetus
Dongusuchus_efremovi
Erythrosuchus_africanus
Garjainia_prima
Proterochampsa_barrionuevoi
Chanaresuchus_bonapartei
Tropidosuchus_romeri
Euparkeria_capensis
Turfanosuchus_shageduensis
Halazhaisuchus_qiaoensis
Osmolskina_czatkowicensis
Turfanosuchus_dabanensis
Gracilisuchus_stipanicicorum
Yonghesuchus_sangbiensis
Ticinosuchus_ferox
Qianosuchus_mixtus
Effigia_okeeffeae
Xilousuchus_sapingensis
Arizonasaurus_babbitti
Combined_Prestosuchus
Saurosuchus_galilei
Batrachotomus_kuperferzellensis
Fasolasuchus_tenax
Rauisuchus_tiradentes
Postosuchus_kirkpatricki
Dromicosuchus_grallator
Sphenosuchus_acutus
Ornithosuchus_longidens
Riojasuchus_tenuisceps
Revueltosaurus_callenderi
Aetosaurus_ferratus
Stagonolepis_robertsoni
Longosuchus_meadei
Eudimorphodon_ranzii
Dimorphodon_macronyx
Lagerpeton_chanarensis
Marasuchus_lilloensis
Lewisuchus_Pseudolagosuchus
Asilisaurus_kongwe
Silesaurus_opolensis
Heterodontosaurus_tucki
Lesothosaurus_diagnosticus
Herrerasaurus_ischigualastensis
Eoraptor_lunensis
Coelophysis_bauri
Saturnalia_tupiniquim
Plateosaurus_engelhardti
Efraasia_minor
Archeopelta_arborensis
Doswellia_kaltenbachi
Jaxtasuchus_salomoni

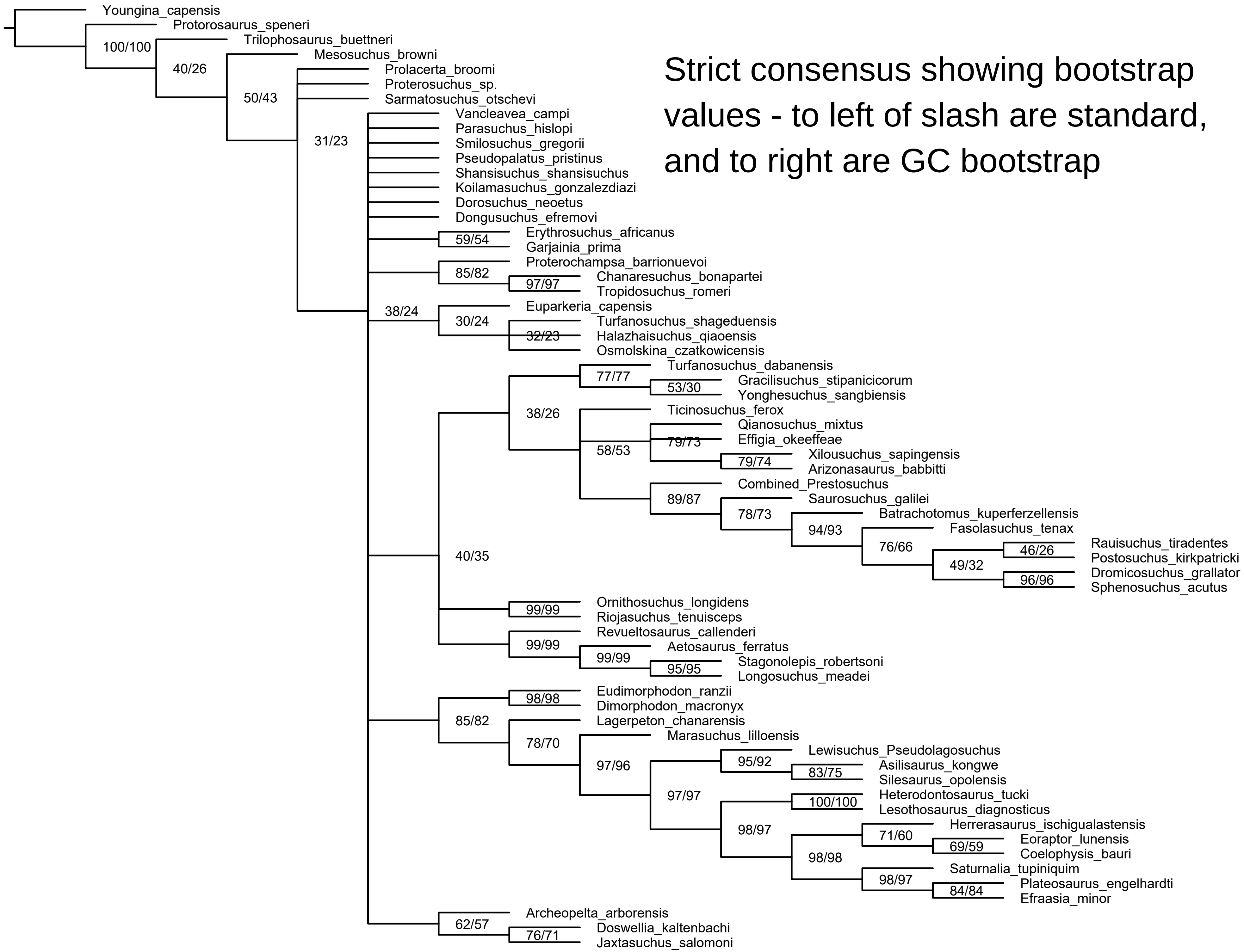

Strict consensus showing bootstrap values - to left of slash are standard, and to right are GC bootstrap

Strict consensus showing Bremer support (=decay indices)

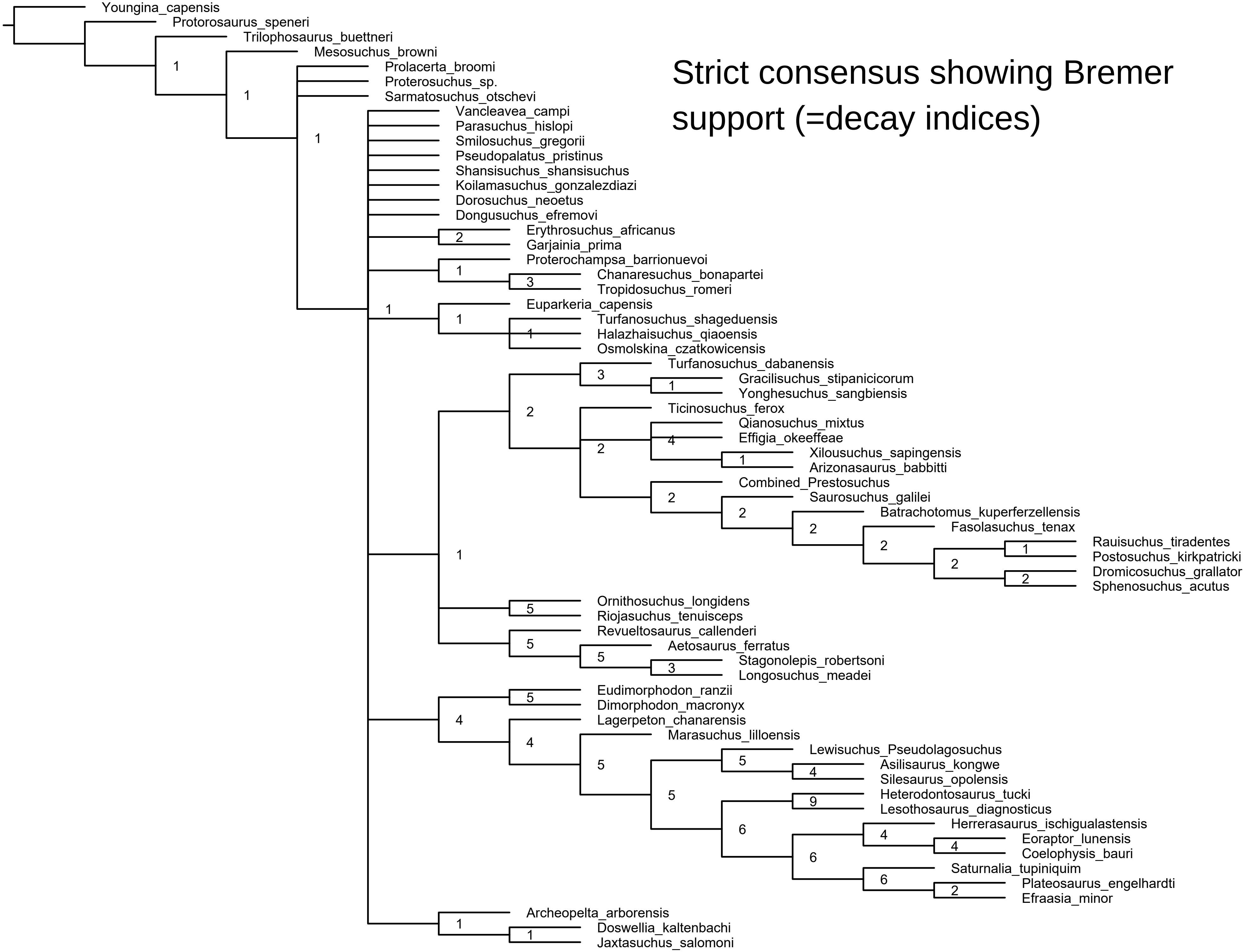

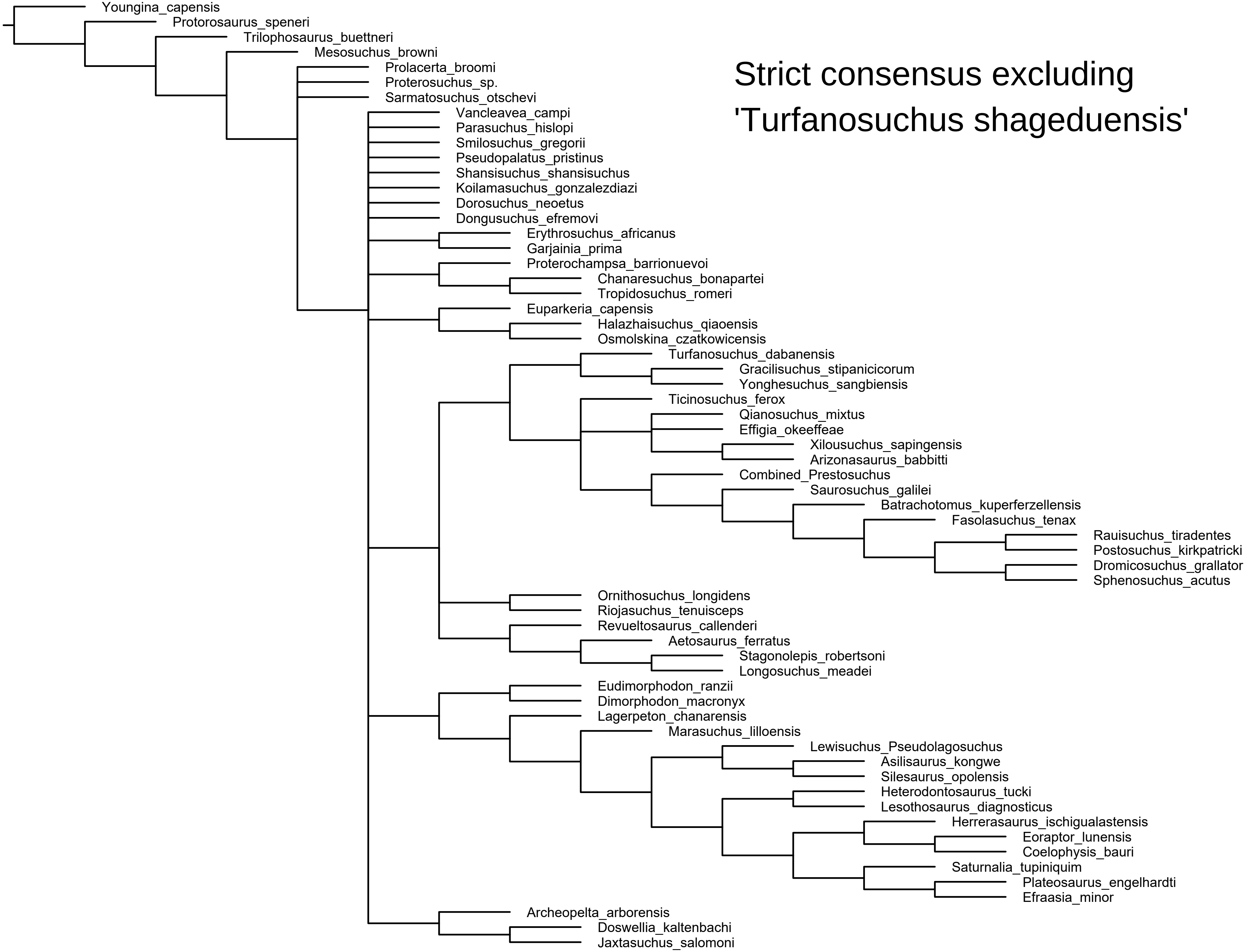

Strict consensus excluding 'Turfanosuchus shageduensis'

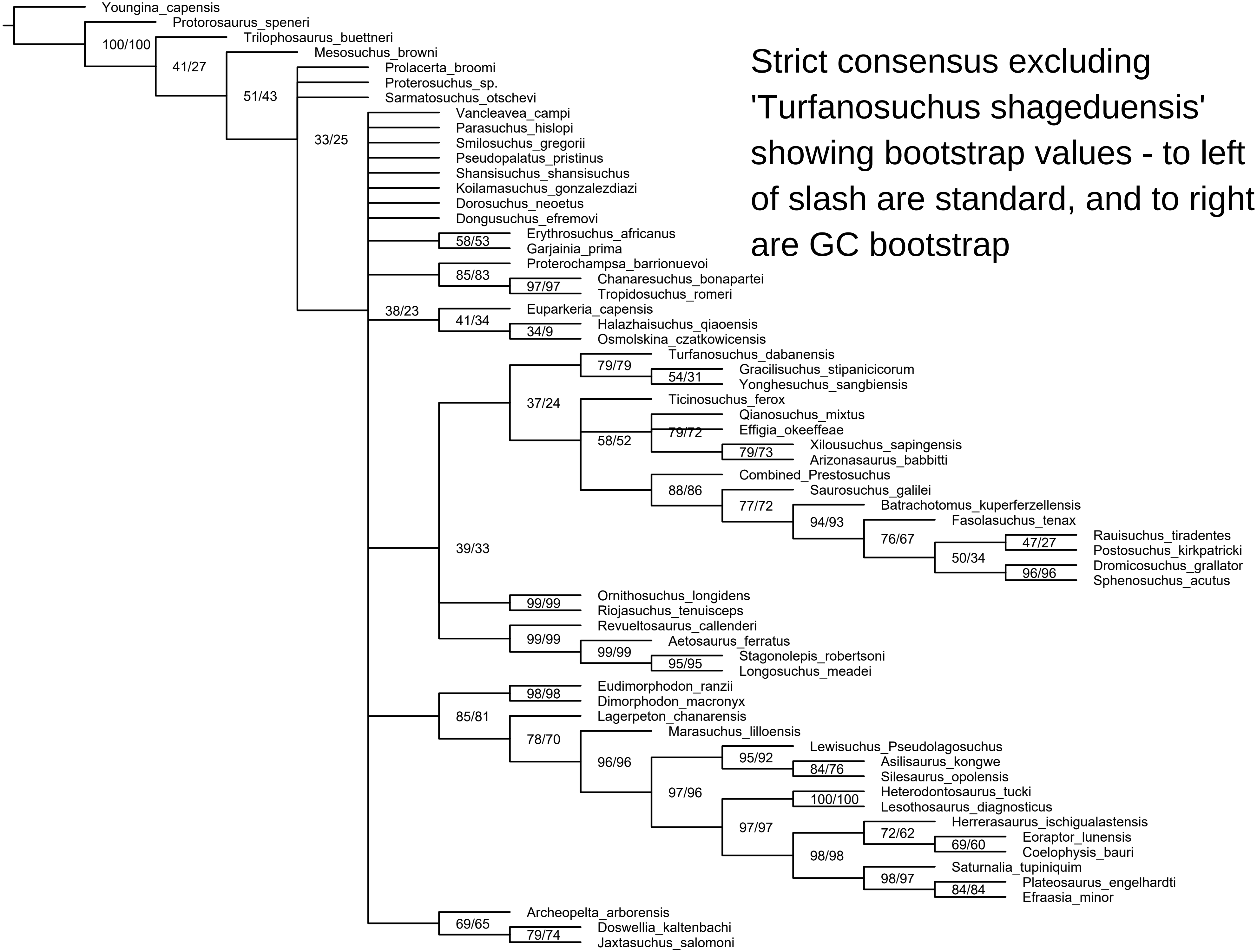

Strict consensus excluding 'Turfanosuchus shageduensis' showing bootstrap values - to left of slash are standard, and to right are GC bootstrap

Strict consensus excluding 'Turfanosuchus shageduensis' showing Bremer support (=decay indices)

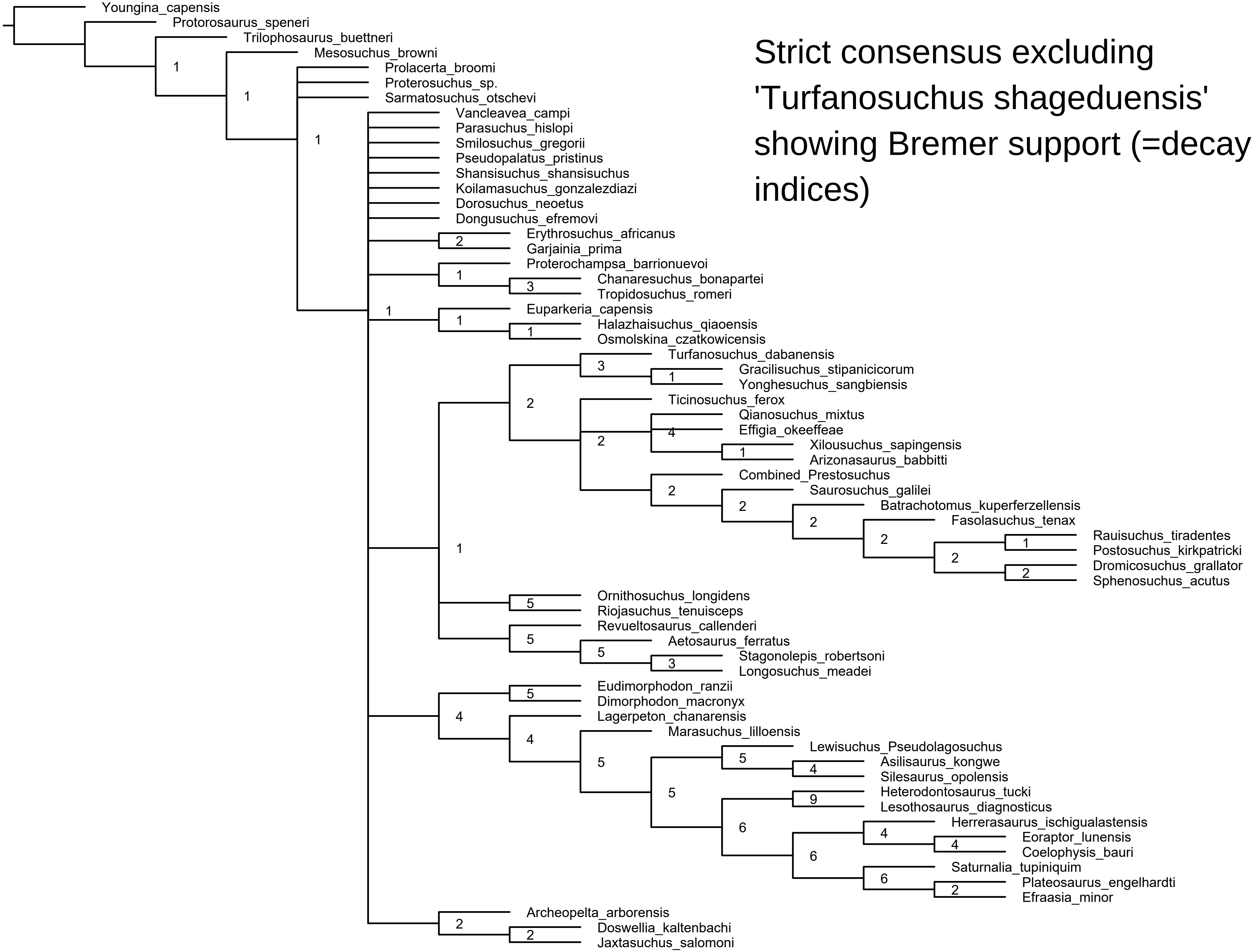

Optimisation of characters relating to palatal dentition onto strict consensus tree

All using modified version of Sookias 2016 – i.e. matrix provided with this work

Optimised using squared change parsimony in Mesquite 3.2

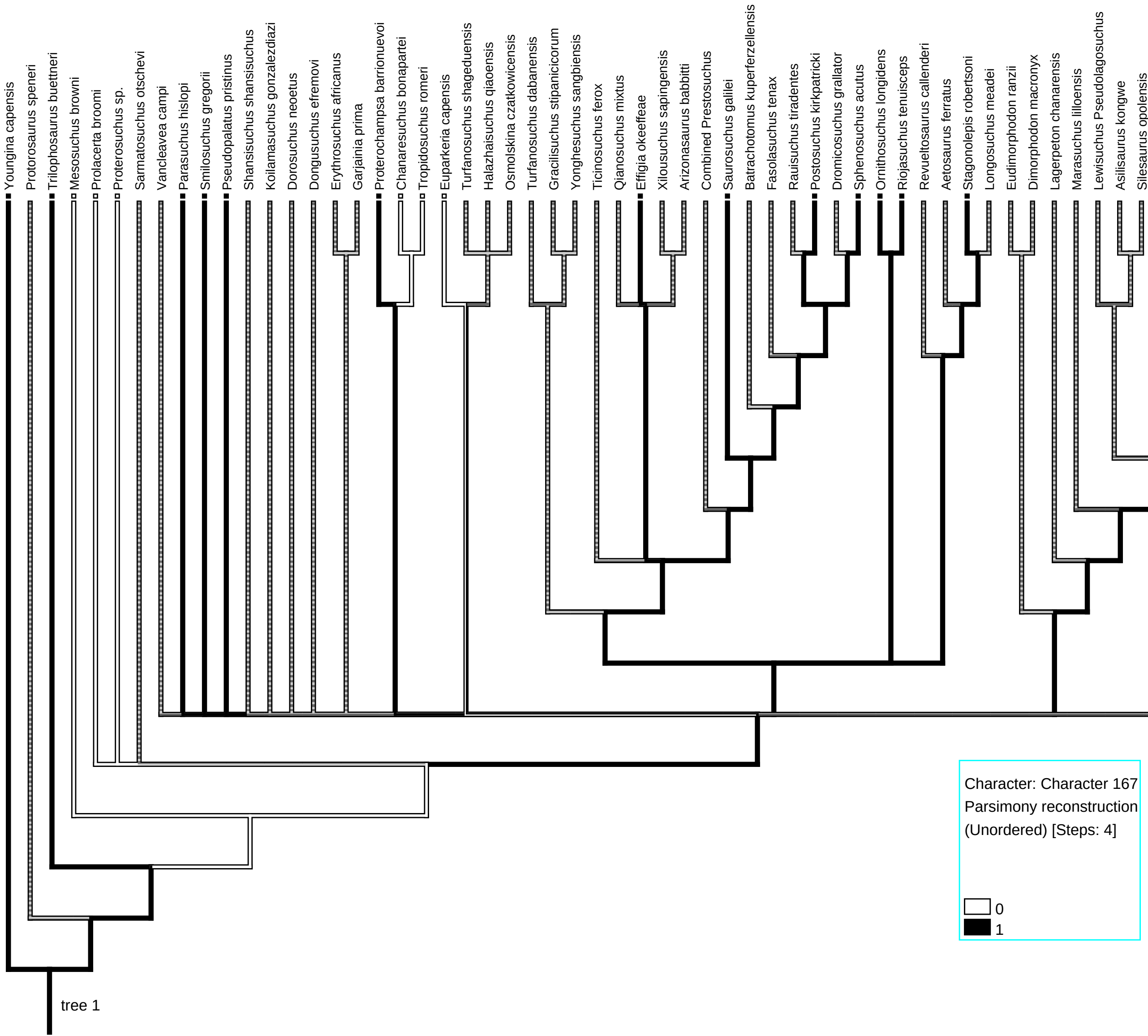

167. Vomer, dentition

(0) present

(1) absent

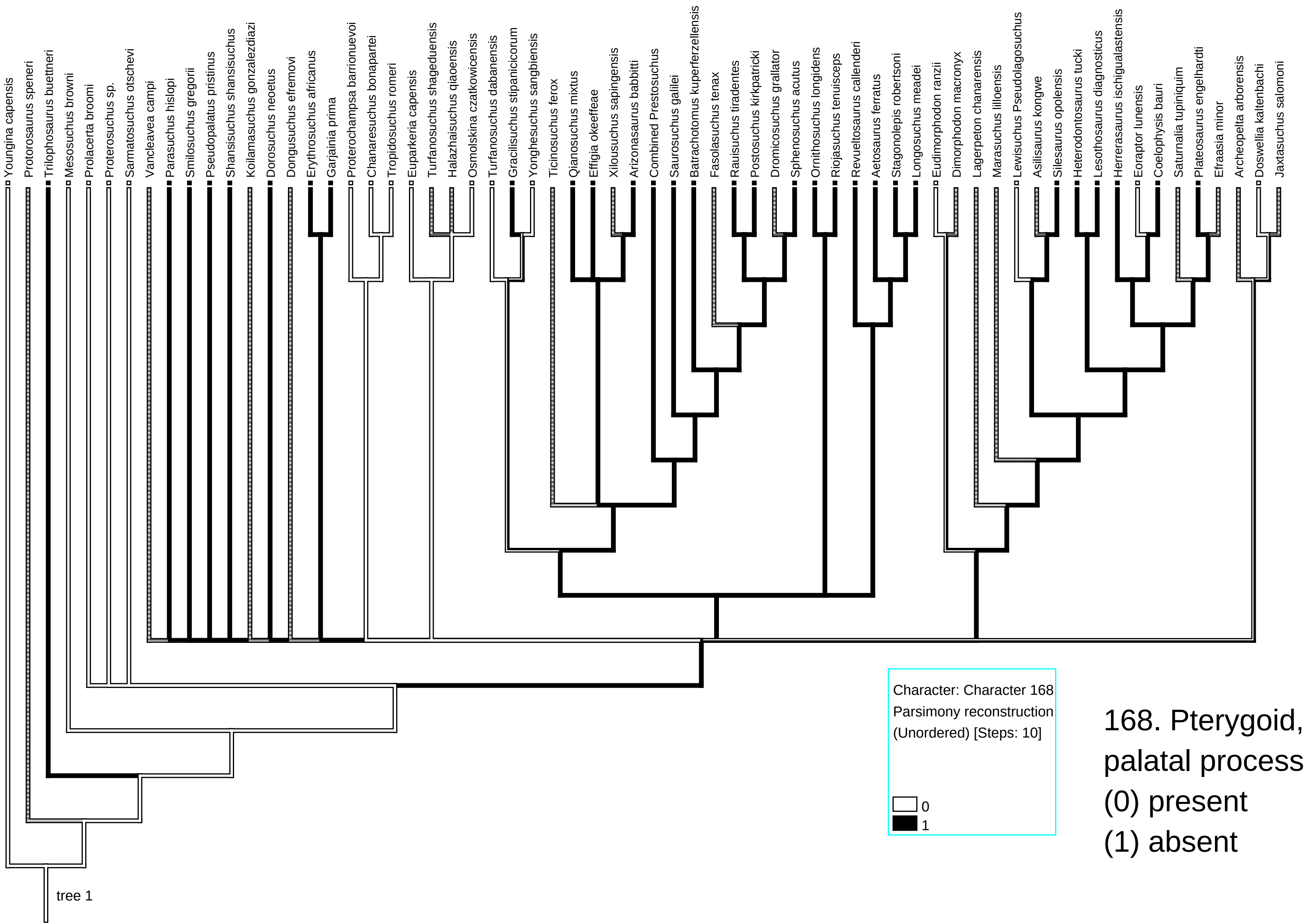

168. Pterygoid, teeth, on palatal process:

(0) present

(1) absent

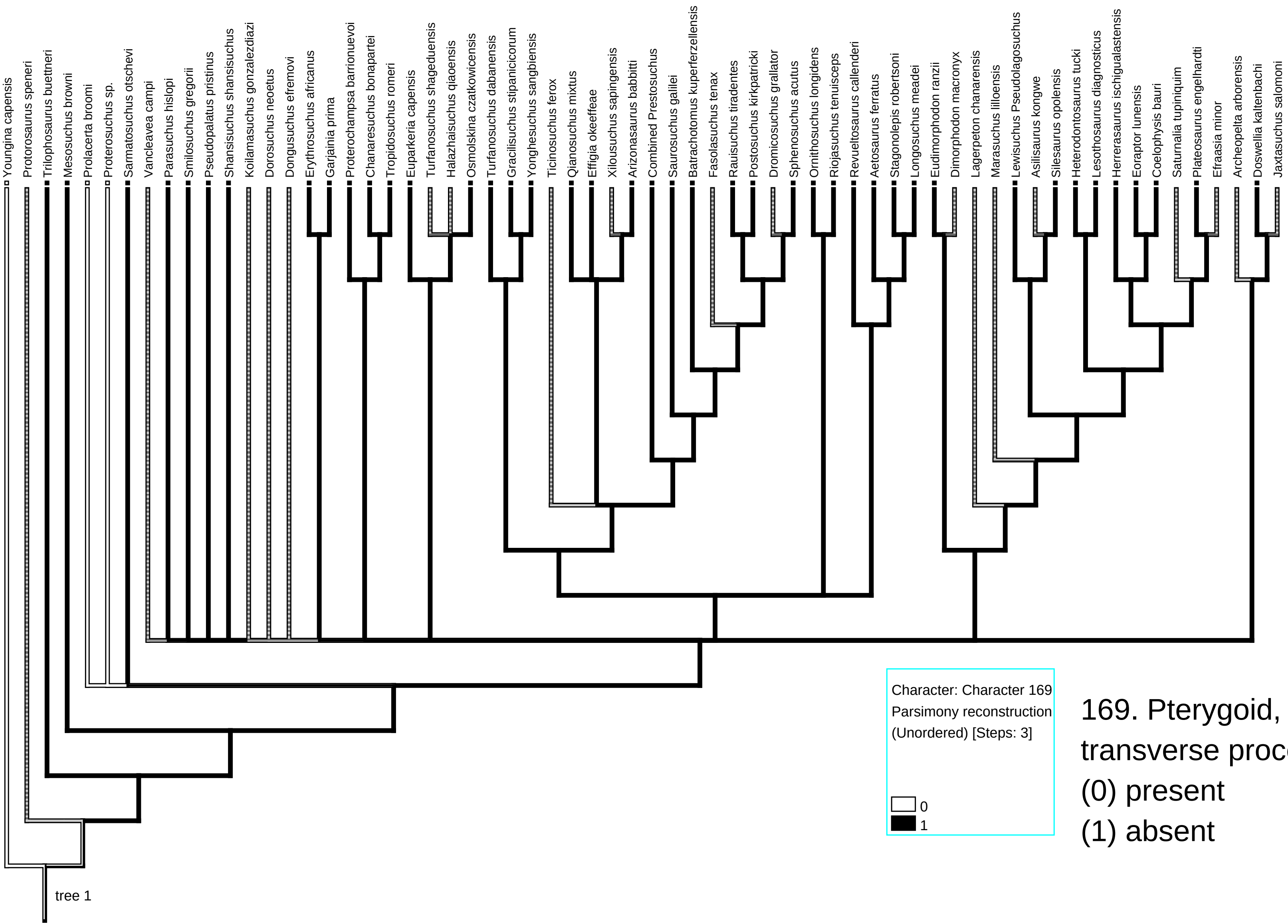

Character: Character 169
Parsimony reconstruction
(Unordered) [Steps: 3]

0
1

169. Pterygoid, teeth, on transverse process:
(0) present
(1) absent

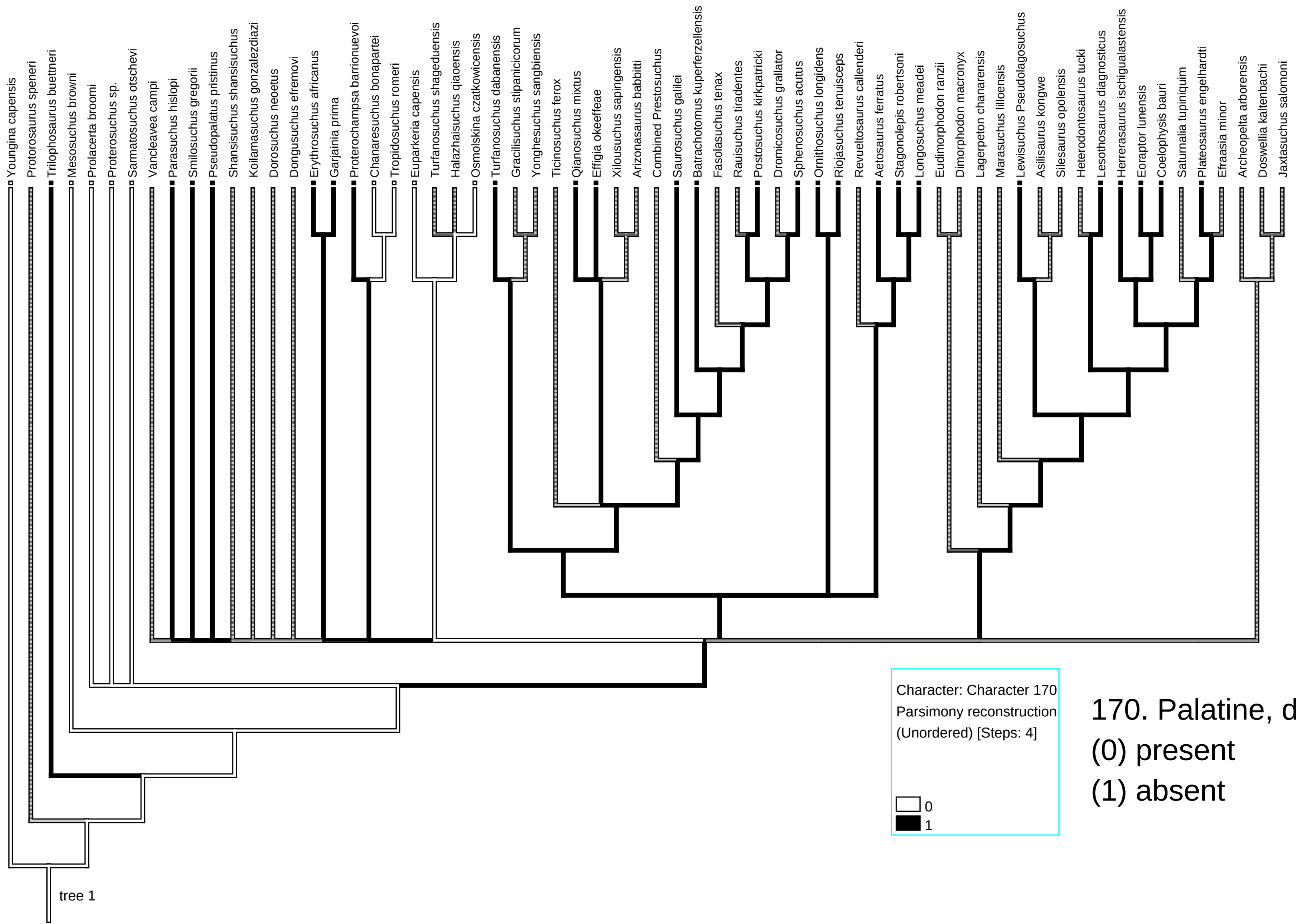

170. Palatine, dentition:

(0) present

(1) absent

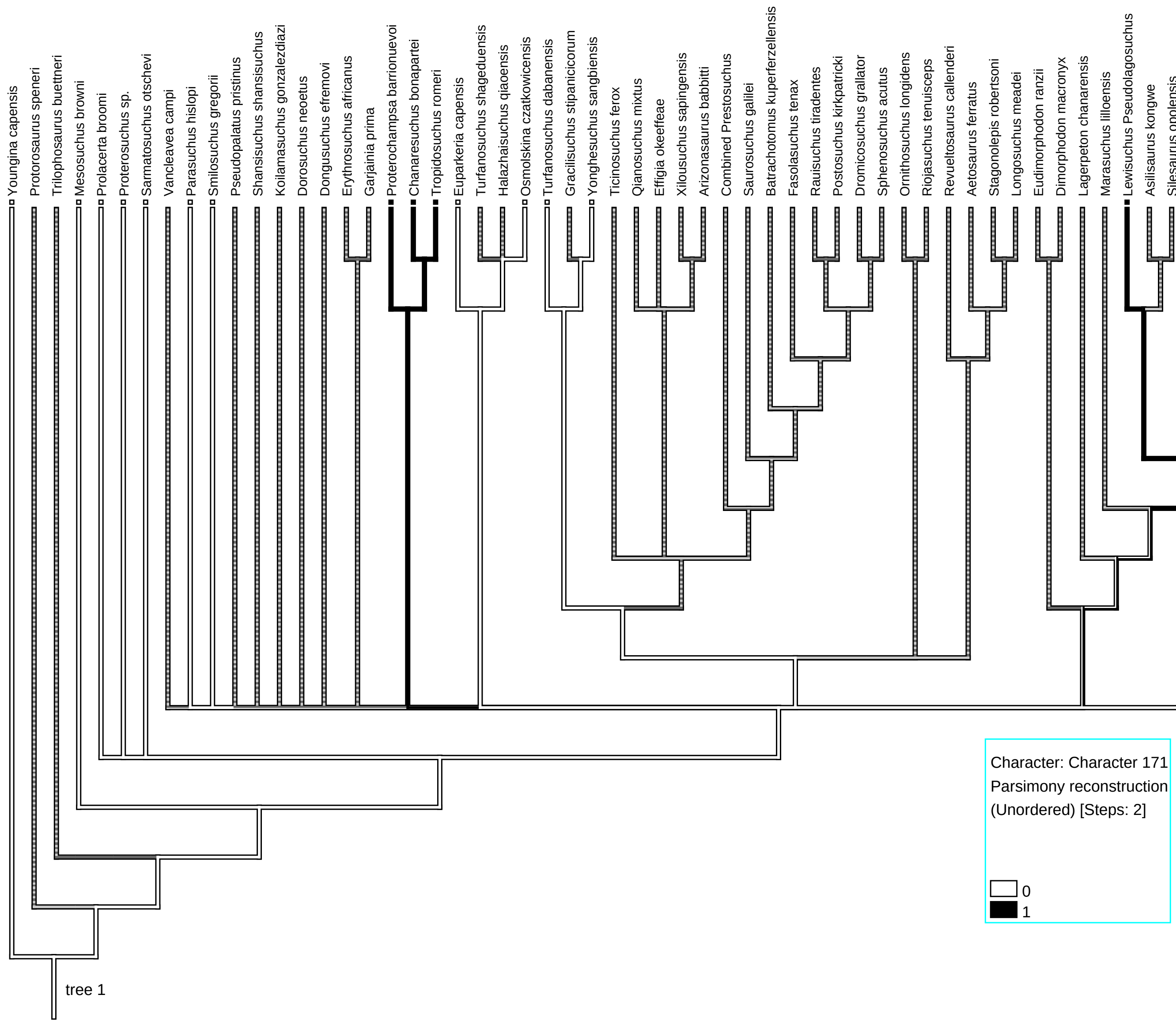

171. Pterygoid/palatine, dentition, attachment
(0) attached to surface of bone
(1) inserted into alveoli
